# Utilization of nurse-sensitive indicators in nursing practice: A comparative qualitative study in German and U.S. hospitals

**DOI:** 10.1016/j.ijnsa.2026.100682

**Published:** 2026-09-18

**Authors:** Carolin Gurisch, Joan Kleine, Julia Köppen, Claudia B. Maier

**Affiliations:** a Department of Healthcare Management, Technische Universität Berlin, Straße des 17. Juni 135, 10623, Berlin, Germany; b Department of Health Sciences, BQS Institut für Qualität und Patientensicherheit GmbH, Wendenstraße 375, 20537, Hamburg, Germany; c School of Public Health, Bielefeld University, Universitätsstraße 25, 33615, Bielefeld, Germany

**Keywords:** Nurse-sensitive indicators, Hospitals, Nursing, Quality improvement, Magnet

## Abstract

**Background:**

Nurse-sensitive indicators represent data that can be used in a learning health system to systematically generate knowledge, identify opportunities to improve nursing practice and enhance patient care.

**Aim:**

To analyze and compare the utilization of nurse-sensitive indicators in nursing practice between selected hospitals from Germany and the U.S.

**Method:**

A qualitative study was conducted, comprising 17 semi-structured interviews with 19 nurses carried out from June to September 2025. Purposive sampling was applied, including nine participants from four hospitals in Germany that were part of the Magnet4Europe study, and ten participants from four U.S. Magnet® hospitals. The interview guide followed the cycles of a learning health system. Data were analyzed using qualitative content analysis according to Mayring. The reporting adhered to the standards for reporting qualitative research (SRQR). The study was not registered.

**Results:**

Three themes and eight subthemes emerged. First, differences were observed in the scope of nurse-sensitive indicators collected, with interviewees from U.S. hospitals reporting a broader range and higher assessed documentation quality. Second, access to and internal structures of working with nurse-sensitive indicators differed between countries. Interviewees from U.S. hospitals described systematic data transparency, clinical roles for data analysis, and structured data discussions across all organizational levels. Interviewees from German hospitals reported sharing data internally with more restricted access, diverse analytical roles, and data discussions mainly within unit teams. All interviewees recognized opportunities to improve nursing care through trends and benchmarking, with U.S. hospitals placing a stronger emphasis on quantitative data and evidence-based practice. Third, interviewees from U.S. hospitals explained standardized evaluation and extensive recognition processes of success, while interviewees from German hospitals reported unstructured or absent evaluation processes and a need to strengthen recognition of achievements.

**Discussion:**

A systematic utilization of nurse-sensitive indicators was demonstrated by the interviewees from U.S. hospitals, whereas interviewees from German hospitals reported a high divergence in their utilization, indicating potential to strengthen their use in the future. Further research is needed to identify enabling factors and barriers for integrating nurse-sensitive indicators into nursing practice.

**What is already known**

– Nurse-sensitive indicators represent an instrument for quality assurance and quality improvement in nursing practice.
– The framework of a learning health system supports the strategic use of data to drive continuous improvements in practice.
– The Magnet® model emphasizes the systematic use of data to guide nursing practice and organizational development.

**What this paper adds**

– A comparative analysis of how nurse-sensitive indicators are actually used to support data-driven improvements in everyday nursing practice in selected hospitals in Germany and the U.S. that are either implementing or have implemented the Magnet® model.
– The findings reveal that the German and U.S. hospitals are in different stages of using nurse-sensitive indicators within the three cycles of a learning health system. While U.S. Magnet® hospitals demonstrated a systematic approach, the potential for German hospitals to further enhance the use of nurse-sensitive indicators for quality improvement is highlighted.

## 1 Introduction

Hospitals around the world produce large amounts of data, one driver of which is digitalization (Advisory Council on the Assessment of Developments in the Health Care System, 2021). Based on an estimation of the World Economic Forum, around 97% of the data goes unused (World Economic Forum, 2019). The concept of a learning healthcare system supports the systematic use of data. Although there is no uniform definition, the description by the Institute of Medicine is widely acknowledged (Friedman et al., 2017). They define a learning healthcare system as a continuous improvement process, where science, informatics and culture are aligned, best practices applied, patients and their families actively integrated into decision-making, and data continuously collected (Institute of Medicine, 2013). Three learning cycles can be differentiated within a learning healthcare system: generating knowledge from data (data to knowledge), applying that knowledge in practice to drive improvements (knowledge to performance) and collecting performance data to generate new knowledge (performance to data) (Friedman et al., 2017).

With nurses being the largest workforce in the healthcare sector (World Health Organization, 2025), it is essential that they use existing quality data to engage in quality assurance (Easter and Tamburri, 2018). Nurse-sensitive indicators, which reflect changes in patient outcomes resulting from nursing care, can therefore be used to monitor and improve nursing practice and patient care (Oner et al., 2021). Internationally, the terminology is used inconsistently (Afaneh et al., 2021). A systematic literature review on nurse-sensitive indicators showed their broad scope and varying adaptation across countries (Oner et al., 2021).

In Germany, there is a mandatory requirement by law for nationwide data collection of one nurse-sensitive indicator, namely on hospital-acquired pressure injuries (IQTIG, 2025). A qualitative analysis on nurse-sensitive quality indicators in 2020 found limited existence of nurse-sensitive data in German hospitals, with hospital-acquired pressure injuries and falls being the indicators collected most often (Maier et al., 2024). In the United States (U.S.), there is no federal legal requirement for hospitals to collect nurse-sensitive indicators, although some states require hospitals to report specific nurse-sensitive indicators as part of quality assurance (e.g., Texas Health and Safety Code § 98.1045). Additionally, collecting and reporting nurse-sensitive indicators are required when participating in specific insurance programs or accreditations. For instance, the Centers for Medicare & Medicaid Services operate the Hospital-Acquired Condition Reduction Program. This program reduces payments to general hospitals treating Medicare patients if they fall within the 25% of hospitals with the highest rates of hospital-acquired conditions (CMS.gov, 2025). Moreover, hospitals can voluntarily participate in the National Database of Nursing Quality Indicators (NDNQI), managed by Press Ganey, which offers the opportunity to benchmark >250 nurse-sensitive indicators (PressGaney, 2025).

Nurse-sensitive indicators provide an important link between the concepts of evidence-based practice, nursing research, and quality improvement by measuring the outcome quality of nursing to inform clinical practice, research, and quality improvement. Evidence-based nursing practice is a problem-solving approach to clinical decision-making that combines current scientific evidence with clinical expertise and knowledge generated through outcomes management and quality improvement initiatives (Mazurek Melnyk and Fineout-Overholt, 2023). As the foundation of evidence-based nursing practice, research plays a crucial role (Powers, 2020), yet a gap between research and practice exists in nursing in Germany. A cross-sectional study of 55 German hospitals with >500 beds from 2016 concluded that nursing research was not a firmly established structural component in the majority of German hospitals surveyed (Horbach et al., 2016). Focusing on German university hospitals, a cross-sectional study of 31 nursing scientists found that half of them were engaged in research projects and spent about 25% of their time on those projects, primarily in qualitative research (Seidlein et al., 2024). Reported hurdles included limited funding, time constraints, and challenges in obtaining ethics approval (ibid.). A cross-sectional study from 2020, including 181 nursing researchers from U.S. Magnet® hospitals, showed that >90% of the participants integrated findings from nursing research in clinical practice to improve quality of care and used an evidence-based practice model (Speroni et al., 2020).

The Magnet Recognition Program (Magnet®) from the American Nurses Credentialing Center aims at recognizing hospitals worldwide for their excellence in delivering nursing care (ANCC, 2025). Its underlying model includes five components, including ‘New Knowledge, Innovation, & Improvements’ and ‘Empirical Outcomes’ (ibid.). Within the component ‘New Knowledge, Innovation, & Improvements’ it is emphasized that nursing practice and patient care should continuously integrate current evidence, research findings, and innovative approaches (ibid.). The component ‘Empirical Outcomes’ requires hospitals to collect nurse-sensitive indicators and compare those to a national benchmark (ibid.).

The Magnet4Europe project (2020–2024) included 60 hospitals from six European countries (Sermeus et al., 2022), with 21 German hospitals participating (Magnet4Europe, 2025). The aim was to support hospitals in implementing the Magnet® model within their organizations (Sermeus et al., 2022). Each European hospital was assigned a U.S. Magnet® hospital as twinning partner (ibid.). The twinning process aimed at transferring experiences and learning from each other (ibid.).

There remains a gap in understanding if and to what extent nurse-sensitive indicators are utilized within German hospitals and what differences exist compared to U.S. hospitals. This study conducted a comparative analysis between U.S. Magnet® hospitals and German hospitals, implementing the Magnet® model, on how the use of nurse-sensitive indicators is integrated into hospital nursing practice to foster data-driven improvements in patient care.

## 2 Method

### 2.1 Study design

A qualitative study was conducted. The study’s reporting follows the standards for reporting qualitative research (SRQR, appendix A) (O’Brien et al., 2014).

### 2.2 Study setting

Purposive sampling was applied to select four German and four U.S. hospitals for data collection. Hospitals from Germany were chosen if they were participants in the Magnet4Europe study, given the importance of empirical outcome data in the Magnet® model (ANCC, 2025). Hospitals from the U.S. were chosen if they held Magnet® status and had a German twinning partner during the Magnet4Europe study. A key informant was contacted in each German hospital and asked to forward the interview request to nurses, working in clinical practice, quality management, nursing practice development or nursing management, with the aim to question one to three nurses from each hospital. Interviewees were purposively sampled across different nursing roles to capture a comprehensive range of perspectives on working with nurse-sensitive indicators as all of these roles are either directly involved in working with such data or affected by its implementation. Moreover, they were asked to share the interview request with their U.S. twinning partner, which all of them did. Of the four U.S. hospitals approached this way, two contact persons agreed to the interview invitation and forwarded the interview request up to two additional colleagues. In order to include two more U.S. hospitals in this study that met the inclusion criteria, contact details were obtained from the project website of Magnet4Europe. Another contact was obtained through a researcher who had prior contact with one of the U.S. hospitals participating in Magnet4Europe. Three U.S. hospitals were contacted this way, two responded, agreed to participate and forwarded the interview request to one additional colleague.

### 2.3 Data collection

Data were collected during a three-month period between June and September 2025. Usually one person was interviewed, but in one case, three people were interviewed together upon their request, due to the interviewees’ time constraints. In total, 17 interviews were conducted with 19 interviewees before data saturation was reached (Glaser and Strauss, 2017). All interviews were conducted online. The interviewer was the same person across all interviews. All but one of the interviewees from the German hospitals knew the interviewer beforehand through a different professional context, whereas there had been no prior contact between the interviewees from the U.S. hospitals and the interviewer.

A semi-structured interview guide was applied, based on the three cycles of the framework of a learning health system (Table 1). The table provides an overview of the cycles of the learning health system, the thematic focus of the interview guide, and example questions to enhance study reproducibility.

**Table 1:** Main themes and exemplary questions from the semi-structured interview guide.

Cycle learning health system	Main topics of the interview guide	Examplary interview questions
Data to knowledge	Current status of nurse-sensitive indicators collected, internal structures of working with nurse-sensitive indicators	What patient outcome data, e.g. falls, do you collect at your ward / in your hospital? How are patient outcome data used on your ward / in your hospital?
Knowledge to performance	Hospital structures for identifying improvement potential	What approach is taken to identify measures to improve patient care / increase quality of care based on patient-outcome data? How are the identified measures implemented to (potentially) improve patient care?
Performance to data	Evaluation and dissemination process of implemented measures	What processes exist to check whether measures introduced / implemented to improve patient care have been successful? How are success stories in improving patient care dealt with on your ward / in your hospital?

All interviews were recorded after consent had been provided on the day of the interview. Sociodemographic data were collected following the completion of the audio recording. All data were stored on a password-protected computer with appropriate security measures, including firewall protection. Access was restricted to two authors (CG, JoK).

### 2.4 Data analysis

The recorded interviews were transcribed verbatim using the software Amberscript. In line with data minimization principles, recordings were started only after the collection of identifying information, such as full names and institutional affiliations, and stopped before the collection of sociodemographic data. This approach ensured that only essential identifying information was captured and submitted to Amberscript. Since it was not possible to remove all identifying details prior to submission to Amberscript, full anonymization of the transcripts was applied immediately afterwards during the review of the version provided by Amberscript. Each interviewee was assigned a pseudonym, and all identifiable information was anonymized within the transcripts. The transcripts were not returned to the interviewees. Coding was performed in ATLAS.ti following the qualitative content analysis by Mayring (2022). The coding used a deductive-inductive approach and was split into two steps. First, the questions from the interview guide served as deductive codes. A codebook was developed and each deductive code was defined. Two researchers coded two interviews each based on the codebook. The results were compared, and the descriptions within the codebook refined. Then the remaining interviews were coded deductively by one researcher. Inductive coding was added by analyzing the different aspects within each deductively formed code. Consistency of this process was ensured across all interviews by having one researcher mainly responsible for the coding. The entire coding process was conducted in English. Coding results were discussed in repeated rounds among all researchers ensuring researcher triangulation.

Units of comparison were the interviews with the selected German and U.S. hospitals in their entirety, with interview results being compared based on the codes (Palmberger and Gingrich, 2014).

The description of the research method and the depiction of quotes enhanced the study’s dependability and transferability. Quotes from the interviewees from the German hospitals were translated by one researcher, while another researcher reviewed the translation by comparing it with the original quote.

### 2.5 Ethics

Ethical approval was obtained on 2nd of June 2025 by the University of Bielefeld (2025–211). Interviewees were repeatedly informed about voluntariness of the interview participation and data protection, lastly before the audio recording of the interviews started. Written informed consent was obtained from all interviewees before the interview, encompassing all aspects of data processing and handling.

## 3 Results

### 3.1 Description of interviewees

In total, 17 interviews were conducted with 19 nurses, including clinical nurses, advanced practice nurses, nurse specialists, unit managers, Magnet® coordinators, nursing directors, nurses from the department quality management and nursing practice development. Interviewees were included from four German and four U.S. hospitals. The size of the hospitals varied between 350 and 1200 beds. One to four persons were interviewed per hospital, with most interviewees being female and having on average 21 years of work experience, with 14 considering their position to be a leadership role. All interviewees indicated that they had been actively involved in patient care, and six interviewees reported that they were still involved (Table 2).

**Table 2:** Characteristics of the interviewees’ sample.

	**German hospitals**	**U.S. hospitals**
**Included interviewees**	9 (1 to 3 interviewees from 4 hospitals)	10 (2 to 4 interviewees from 4 hospitals)
**Sex**		
Female	5	10
Male	4	0
**Work experience** (average in years)	22	20
**Leadership position**		
Yes	6	8
No	3	2
**Involved in direct patient care**		
Yes	4	2
No	5	8

### 3.2 Themes and sub-themes

The interview results were summarized into three main themes and eight sub-themes reflecting the cycles of a learning health system: Variety and documentation quality of nurse-sensitive indicators, access to and internal structures of working with nurse-sensitive indicators, and evaluation and dissemination of data-driven improvements of nursing practice (Table 3).

**Table 3:** Themes and sub-themes.

**Themes**	**Sub-themes**	**Description of hospitals from Germany**	**Description of hospitals from the U.S.**
**Variety and documentation quality of nurse-sensitive indicators**	Scope and assessment of nurse-sensitive indicators	Falls and pressure injuries are the most common indicators; expansion of indicator set planned; indicator set rated divergently as suitable as well as incomplete	Comprehensive indicator set; suitable indicator set
	Documentation quality and safety culture	Delays and incomplete documentation; technical challenges affecting data quality; handling of errors	High documentation quality, but underreporting of the indicator ‘assaults on nurses’; just culture
**Access to and internal structures of working with nurse-sensitive indicators**	Data transparency on the units	Role-based access for nurses up to availability on the intranet for all professions	Data transparently displayed on unit dashboards; accessible to all professional groups and patients; content adapted to the target group
	Responsibilities for data analysis	Unit leaders; partially involvement of clinical experts, nursing development, nursing controlling	Clinical experts for indicators; partially systematic involvement of frontline nurses and leadership roles
	Contexts and formats of data discussions	Ranging from regular to no meetings at the unit level, with occasional meetings across different organizational levels	Regular discussions at all organizational levels (unit, division, organization)
	Identifying ideas for improvement	Improvement ideas driven by trends, benchmarking; frontline nurses involved to not involved at all; need to strengthen evidence-based practice	Improvement ideas driven by trends, benchmarking, and continuous quality goals; involvement of frontline nurses; strong hospital-wide support for evidence-based practices
**Evaluation and dissemination of data-driven improvements of nursing practice**	Measuring success of implemented measures	Evaluation processes are often unstructured or not yet conducted	Strong focus on data trends; structured evaluation processes and use of process measures
	Recognizing and sharing successful practices	Verbal and written communication channels used; need to strengthen recognizing and acknowledging success	Strong culture of recognition with verbal, written communication and celebrations; dissemination of best practices across units

#### 3.2.1 Variety and documentation quality of nurse-sensitive indicators

##### 3.2.1.1 Scope and assessment of nurse-sensitive indicators

All interviewees from German hospitals mentioned data collection of hospital-acquired pressure injuries and falls, making these the most common nurse-sensitive indicators to be collected. Half of the interviewees from German hospitals reported ongoing or planned data collections on nosocomial infection rates and named further indicators, such as delirium, with which they planned to expand the indicator set.

*“And that is about the typical topics such as falls, pressure injuries, and nosocomial infections.“ (I10_GER)*

In contrast, all interviewees from U.S. hospitals reported a broader scope. Data collections on infections, falls, hospital-acquired pressure injuries and assaults on nurses were mentioned by more than half of the interviewees. Several other indicators were named inconsistently across interviewees.

*“CLABSI, CAUTI, HAPI, falls, unplanned extubations and PIVs [peripheral intravenous catheters], are kind of our top six that we look at. We also include CDIFF in there. And then there's some new ones that are coming out around delirium and then some staff safety ones.” (I12_US)*

Most U.S. hospital and half of the German hospital interviewees considered the current scope of indicators appropriate, emphasizing their relevance to nursing quality and evidence-based grounding.

*“So you know we can see a lot in these nursing sensitive indicators, are specific to the quality of nursing care. You know, any of the NDNQI ones are ones that have been proven through research.” (I15_US)*

Some interviewees from German hospitals, however, were concerned that expanding indicator sets could overwhelm staff and reduce engagement with data.

*“I think it is […] sufficient at the moment, because I believe that if we add even more indicators now, the nursing staff would be overwhelmed at first.“ (I1_GER)*

Four interviewees of German hospitals nonetheless expressed the need for to expand data collection, noting barriers such as technical difficulties in extracting data from the patient information systems, missing financial incentives for hospitals and a lack of perceived relevance to identify and systematically collect further indicators.

##### 3.2.1.2 Documentation quality and safety culture

All interviewees from German hospitals reported challenges with the documentation quality of nurse-sensitive indicators. This was attributed to a lack of perceived relevance among staff, leading to delayed recording, incomplete and incorrect documentation. Technical barriers contributed to data biases and increased efforts in data preparation.

*„That was due to technical issues, […] is that really an admission finding or is it a nosocomial finding? Or is it a finding that was already reported last week […].“ (I2_GER)*

The handling of errors in German hospitals was mostly described with a negative connotation as the focus was on mistakes and punitive approaches. At the same time, half of the interviewees described a shift to framing data as a supportive tool for improvement and mutual learning.

In contrast, most interviewees from U.S. hospitals assessed the documentation quality as good and explained that many measures had been implemented to ensure consistent documentation. A few interviewees emphasized, however, that there is still room for improvement in the quality of the indicator measuring assaults on nurses, as it is considered underreported.

*“And I know that our assaults on nursing rates are definitely underreported […]. And we've tried to talk to the nurses and, you know, reinforce that you should report these.” (I15_US)*

All interviewees from U.S. hospitals reported that a positive and learning-oriented just culture is predominant making staff feel safe reporting all events. The value of data collection is highlighted, emphasizing that it is a supportive tool. Best practices are shared to learn from one another and support each other, with a focus on the positive instead of being punitive. Errors are never addressed individually; the team is the focus.

*“We have done a ton of work around our safety culture and making sure that staff are empowered to speak up and empowered to report. […] It's all coming from a system perspective of there are holes in our system that we need to fix and never punitive to the individual.” (I12_US)*

#### 3.2.2 Access to and internal structures of working with nurse-sensitive indicators

##### 3.2.2.1 Data transparency on the units

All German hospitals interviewees reported that unit leaders receive results on nurse-sensitive indicators and are responsible for sharing them with their teams. Dissemination practice varied from presenting data in team meetings or displaying paper-based charts on the units. However, interviewees emphasized that data is not consistently shared.

*„At first, nursing managers had access to it so they could pass it on to the nurses as they saw fit. Now many nurses also have access to it themselves.“ (I1_GER)*

U.S. hospital interviewees reported generally high data transparency and stated that quality boards are used on the units to display data, mostly unit leaders were reported as being responsible for updating them. The quality boards on the units guarantee that data are accessible to nursing teams, other professional groups, and patients and their families.

*“[…] and available up in a place where everybody can see it, including patients and family members, because we want them to see that and ask questions. So that's on the unit. It's on the unit level. You'll see it posted everywhere […].” (I15_US)*

To ensure that the real-time status of nurse-sensitive indicators is quickly apparent, nearly all interviewees from U.S. hospitals reported on displaying “days since last”, indicating how many days had passed since the last event, e.g. a fall, occurred.

*“[…] if what we have learned from the frontline staff is, is they don't really care about a rate number. […] they care about the count, how many? […] And how many days has it been since our last? […] They just want it simplified.” (I12_US)*

Some interviewees from both countries explained that data are also available through online dashboards or monitors, sometimes in real time, and occasionally published on the hospital intranet. Access to these sources ranged from specific roles, such as unit leaders or quality experts to all nurses interested in viewing the data.

Across both countries, the shared data displays included, to varying extents, counts, rates, trends, benchmarks, and when available rankings. Some also added information on preventive measures taken and short explanatory notes to help staff interpret the data. Some interviewees from U.S. hospitals emphasized tailoring the data presentation to different audiences to ensure relevance without overwhelming staff.

##### 3.2.2.2 Responsibilities for data analysis

Most interviewees from German hospitals reported that unit leaders were, or should be, responsible for analyzing nurse-sensitive indicator data. Some also mentioned nursing directors and heads of nursing departments being involved in analyzing the data.

*„And then I [unit manager] always look into the data. What developed within [hospital], what have they [patients] brought along, I evaluate that […].“ (I13_GER)*

In addition, about half of the interviewees from German hospitals mentioned further roles involved in data analysis, either already in place or planned for the future. These included nurses assigned to the analysis of one or more nurse-sensitive indicators, advanced practice nurses, representatives from the nursing development and controlling department.

All U.S. hospital interviewees described having expert roles, so-called champions, being responsible for analyzing one or more nurse-sensitive indicators. These roles were reported to exist for the organization, servicelines, divisions, and the unit levels.

*“But that's been historically our set up or our makeup that's worked for us is to have a person sort of be an expert in a couple, one or two indicators to help the whole hospital achieve success.” (I7_US)*

Half of the interviewees from the U.S. hospitals described frontline nurses as being involved in analyzing nurse-sensitive indicators, either by leading unit-based quality councils or by taking on quality-related tasks for all relevant nurse-sensitive indicators within their team.

*“So every single unit would have a quality council that meets monthly. And they would have a unit based quality chair [….] one of the staff nurses would help lead that with her clinical director or manager.” (I15_US)*

Some interviewees also described leadership positions being integrated in data analysis, such as unit leaders, leadership teams from each division, serviceline, or the hospital level.

*“It is the responsibility of the unit leadership, yes, to take their quality metrics and to use them to drive change. And they're actually held accountable to that as a part of their annual evaluations.” (I15_US)*

##### 3.2.2.3 Contexts and formats of data discussions

Discussing nurse-sensitive indicators during team meetings at the unit level was mentioned by all interviewees from German hospitals. While half of them described these discussions as regular practice, others indicated that such structures were still in development.

*“Basically, it's just the case that […] this is passed on during team meetings. But I also know that, unfortunately, this doesn't happen across the hospital.” (I11_GER)*

Similarly, about half of the interviewees from German hospitals explained that the data is discussed among leadership. Reports ranged from one-time exchanges to annual strategic meetings and monthly meetings. Some interviewees also described ad hoc discussions between representatives from nursing development and unit leaders as well as exchanges within the nursing directorate in response to specific issues. Moreover, a few interviewees reported data discussions taking place (semi-)annually among nursing experts of specific indicators, with an external network, including hospitals from the same hospital system or participants in the same benchmark, and specific occasions are used to present the data, such as self-organized congresses.

All U.S. hospital interviewees emphasized that the nurse-sensitive indicators are discussed at multiple levels within their hospitals. They reported organization-wide committees for each nurse-sensitive indicator meeting monthly to talk about them. Some interviewees also described having divisional quality councils that focus on all indicators relevant to their divisions.

*“And then we have […] evidence-based practice groups for each of the nursing-sensitive indicators. So falls, CAUTI, CLABSI, and they discuss their specific indicator and try to continue to make improvements on, like nursing practice across the hospital.” (I16_US)*

At the unit level, regular team meetings are held, during which unit leaders discuss the indicators with their teams. Additionally, about half of the interviewees reported that their units have their own quality teams that regularly review and discuss unit-specific indicators. One interviewee highlighted that these meetings are led by frontline nurses whenever possible. Some interviewees also mentioned that safety huddles take place either daily or immediately after an event occurs, allowing direct discussion between nurses and team leaders.

*“[…] then also we do a post fall huddle. And so that is a little group with the charge nurse that says, hey, this is the fall that we had. These were the fall interventions that we had in place. These were the fall interventions that were not in place at the time of the fall. You know, where are our areas that we can improve upon for the next time?” (I18_US)*

Furthermore, the interviewees described reviewing the indicators among the unit leaders, department leadership, multidisciplinary leadership teams and hospital directors. According to the interviewees these meetings can take place from daily to monthly, with urgent sessions happening when necessary.

“And every morning we have a […] safety call. And basically all of the directors check in and like they discuss other things, but they also discuss if they're having like an assault, like a patient that's combative as well as patient falls.” (I12_US)

In one case, regular discussion about the indicators also occurs across hospitals from the same hospital system.

##### 3.2.2.4 Identifying ideas for improvement

Interviewees from both countries explained identifying opportunities for improvement through data trends, reflecting patterns such as increasing fall rates, and by comparing results with internal and external benchmarks, meaning comparing nurse-sensitive indicators over time, across departments, and with data from other hospitals or literature. Some interviewees from German hospitals explained that trends are not necessarily derived from quantitative data but may also be recognized based on subjective assessments. In addition, specific events may trigger data analysis aimed at identifying areas for improvement, for example, the occurrence of pressure injuries of category three or higher, or unusual patterns within a patient population such as a disproportionately high incidence of an event among younger patients.

Some interviewees from U.S. hospitals highlighted their continuous improvement efforts being embedded in the hospital’s overarching goals and quality objectives.

*“I don't know if there's a trigger for it, but I will say every year our hospital goals are always harder and harder. So, like, there's never a time where you're like, oh, this is great. We can do nothing. Everything's good. Like we're always trying to improve […].” (I16_US)*

Likewise, all interviewees emphasized the importance of involving frontline nurses in the process of generating new ideas, both to developing an understanding of how processes are carried out in practice and to adapting these processes accordingly. While this is common practice in the U.S. hospitals included in this study, it ranges from occasional contributions by frontline staff to a complete lack of participation in the German hospitals.

*“And then you want to get people who are doing the daily work who can actually, you know, contribute to the conversation and say, yes, I know we're supposed to be doing this, but here's what's actually happening […] they give us context to what's actually happening, and then they help us design best practice.” (I15_US)*

*“I miss that sometimes too. That my colleagues are genuinely interested in their field of work and are committed to it […].“ (I11_GER)*

Several interviewees from the German hospitals reported that the systematic integration of evidence-based best practices, which refers to the integration of scientific evidence, remained limited but represented an important future goal. The interviewees emphasized the importance of access to scientific literature as a first step, while reporting differing practices, ranging from literature searches conducted by designated roles to none at all due to limited access within the hospital. Specific roles were seen as responsible for promoting evidence-based work, with national expert standards in nursing providing an overarching framework.

*“I think one important aspect of the multiplier structure would be the advanced practice nurses. […]. Otherwise, putting scientific evidence into practice is also a major challenge.“ (I8_GER)*

*“Of course, we also use the expert standards and the guidelines contained in there as a basic framework.“ (I10_GER)*

Half of the interviewees from U.S. hospitals emphasized that hospital-wide policies and standards mandate evidence-based practice. In addition, some described initiatives to actively enable staff to identify best practices, supported through structured training programs. Nearly all interviewees underlined the central role of literature, noting not only access to databases and journals but also the joint review of current findings.

*“And then we look at what are the best practices. So we train everybody as nurse residents and we try to reinforce, you know, using your resources to look to see what best practice is.” (I15_US)*

#### 3.2.3 Data-driven improvements of nursing practice

##### 3.2.3.1 Measuring success of implemented measures

Nearly all interviewees explained that the success of a new idea or intervention was primarily assessed by the reduction of incidence rates. In addition, a decrease in the severity of events for the targeted indicators, employee satisfaction and economic effects were also mentioned to be considered. While the interviewees from U.S. hospitals emphasized defining success from the outset and stressed the importance of relying on measurable outcomes, interviewees from German hospitals explained relying either on quantitative data or drawing on subjective impressions.

*“[…], if you're setting up a pilot, you would have to make sure that you're setting up the pilot for a long enough period of time where you could see outcomes. So if it's like central line rounding, did that improve outcomes? You would need like several months of data. […] if it's working, if it's sustainable […].” (I16_US)*

According to the interviewees from German hospitals the degree of standardization in evaluation varied. While some reported not having standardized processes and rather relying on individual approaches, others had no evaluation processes in place at all.

*“But that people then actually take the consistent evaluation step, I often don’t see that. Or things are often given up too quickly. […] in nursing, it takes time until everyone has been trained, and then they just say […], ‘it didn’t work’. Yes, because it was applied incorrectly […].” (I9_GER)*

In contrast, all interviewees from U.S. hospitals described following structured evaluation approaches, including planned timelines and ensuring the availability of high-quality baseline data. Some also stressed the importance of involving staff in defining the desired changes and thereby fostering an understanding of the underlying reasons for change and ensuring the sustainability of new ideas or interventions. Half of them highlighted the importance of using process measures to verify whether new processes or interventions were applied correctly and whether those could lead to measurable changes in outcome indicators.

*“And that's where the process measures come into play, because that's how we're measuring the progress on those things. So are we doing the right things? And are we doing them consistently?” (I5_US)*

Two German hospital interviewees described adapting the use of process measures from their U.S. twinning partners and applying it in practice.

##### 3.2.3.2 Recognizing and sharing successful practices

The importance of providing multiple opportunities to share achievements was consistently emphasized by the interviewees from U.S. hospitals, who reported each using a variety of channels to do so. Interviewees from German hospitals reported sharing successes, though not with the same intensity as within U.S. hospitals, to not engaging in any communication yet. Some German hospital interviewees explained the need to learn to recognize success in nursing practice, and that some professional groups have to learn to praise success.

*“The one person who actually gives praise once a year is my boss [nursing director]. […] The ones who really need to learn this are the nursing managers. They need to learn that they should also give praise and not just punish people.“ (I3_GER)*

Through all interviews, reported channels for recognizing success included presentations at internal meetings, newsletters, verbal recognition, implementing successful measures on other units, as well as small tokens of appreciation, such as the inclusion of frontline staff in meetings to present success stories themselves or invitations to conferences.

*“And* t*hen we still celebrate at the units giving lots of shout outs based on, you know, what's going on and who's celebrating what.” (I12_US)*

In addition, the interviewees from U.S. hospitals mentioned integrating success stories into their Magnet® documents, presenting them at scientific conferences, and publishing them. The latter are aims of some German hospitals but have not yet been implemented in practice. Celebrations with teams were also mentioned by some of the U.S. hospital interviewees.

*“So when [name unit] went 100 days with no falls, it was in the fall time, the autumn of the year. And so we celebrated them by having a hot chocolate party.” (I17_US)*

## 4 Discussion

This qualitative study explored the utilization of nurse-sensitive indicators in a selection of German and U.S. hospitals for improving nursing practice and enhancing patient care. While all interviewees recognized the importance of data-driven quality improvement, the findings revealed that the interviewees from four German and four U.S. hospitals were in different stages of using nurse-sensitive indicators within the three cycles of a learning health system. Interviewees from the U.S. hospitals described working within well-established infrastructures with a large indicator set, high data quality and transparency, strategic discussions and use for continuous improvement, supported by structured evaluation processes and extensive recognition of success. In contrast, interviewees from the German hospitals reported a smaller set of nurse-sensitive indicators, challenges with data quality, data use for improvement ranging from established to absent, unstructured or missing evaluation processes, and a need to strengthen the recognition of success. These differences reflect not only varying degrees of digitalization and data culture but also broader structural factors that shape nursing practice in each country.

### 4.1 Variety and documentation quality of nurse-sensitive indicators

In the first phase of a learning health system, ‘data to knowledge’, the availability of data is a precondition for being able to work with it (Friedman et al., 2017). The interviewees from U.S. Magnet® hospitals reported having a larger set of nurse-sensitive indicators than those from German hospitals. Magnet® designation requires hospitals to benchmark four nurse-sensitive indicators for all inpatient units and three nurse-sensitive indicators for all outpatient units (ANCC, 2024). Approximately 97% of the American Magnet® hospitals use NDNQI by Press Ganey (PressGaney, 2025). Hence, the included Magnet® hospitals in this study were required to have a larger indicator set and had access to a wide range of indicators for benchmarking. In contrast, the German hospitals were only legally required to collect data on pressure injuries (IQTIG, 2025).

When asked about the data quality and safety culture, interviewees from the German hospitals reported technical and reporting issues. In a previous study, the Digital Radar score was used to assess the digitalization level of German hospitals on a scale from 0 to 100 points across seven dimensions (Geissler et al., 2024). The results showed that hospitals had an average score of 33 points, with particular challenges in information exchange within hospitals (ibid.), which aligns with the reported difficulties in data preparation and clearing by the interviewees. To measure patient safety culture in hospitals, the Hospital Survey on Patient Safety Culture, by the U.S. Agency for Healthcare Research and Quality, has commonly been used (Sorra and Nieva, 2004). One dimension of this survey is the ‘nonpunitive response to error’ (ibid.). A comparative analysis across Economic Co-operation and Development (OECD) countries, including the U.S. but not Germany, showed that this dimension received low scores compared to other dimensions, with an average of 54% of respondents giving a positive response (de Bienassis and Klazinga, 2024). Managers tended to rate this dimension more positively than nurses or physicians (ibid.). Data collected in a German university hospital indicated an average of 52% agreement with non-punitive approaches to mistakes (Wagner et al., 2018). Hence, this appears to be a field with general potential for improvement in both U.S. and German hospital settings, especially highlighting the potential reported by German interviewees in shifting from a negative, error-focused perspective toward a just culture, with a more positive learning-oriented approach for fostering data quality and thereby patient safety.

The perceived underreporting of the indicator ‘assaults on nurses’ by the interviewees from U.S. hospitals is in line with a cross-sectional survey conducted in Canada in 2019 that analyzed the reasons for underreporting patient violence toward nurses (Lee et al., 2025). Among the reasons given by nurses for not reporting such incidents were lack of leadership support, no changes following the reporting of events, and missing knowledge about how to report them (ibid.). Consequently, raising awareness for this indicator and addressing these barriers can promote reporting violence toward nurses.

### 4.2 Access to and internal structures of working with nurse-sensitive indicators

For the phases from ‘data to knowledge’ and ‘knowledge to performance’, interviewees from U.S. hospitals reported that data were discussed across all organizational levels and that data analysis was predominantly conducted by clinical nurses. In contrast, interviewees from German hospitals indicated that data were most often discussed at the team level, with nursing leadership frequently responsible for the analysis. One possible explanation for the more comprehensive use of data in the U.S. may be the greater availability of resources. Already in 2012, a cross-sectional survey including hospitals from 12 European countries and the U.S. showed large variations in staffing levels between hospitals (Aiken et al., 2012). While the patient-to-registered-nurse ratio was 13 in Germany, it was 5.3 in the U.S., indicating better nurse staffing levels in the U.S. compared to Germany (ibid.).

Beyond staffing resources, differences in nurses’ education may help explain why nurse-sensitive indicators are more deeply integrated into clinical nursing practice in the U.S. and why the involvement of frontline staff tends to be more successful. The engagement of nursing staff in quality improvement has been a topic of interest in numerous studies (Alexander et al., 2022; Mulkey, 2021). The Nursing Quality Improvement in Practice tool is a validated instrument designed to measure nurses’ abilities, engagement, beneficial aspects and hurdles related to quality improvement projects (Tschannen et al., 2020). In a cross-sectional survey conducted among nurses in a U.S. hospital, data was collected using this instrument (Tschannen et al., 2021). Analysis revealed that nurses’ skills and attitudes toward quality improvements projects were significantly influenced by their educational level (ibid.). Likewise, an integrative review exploring the benefits and challenges of nurses’ participation in quality improvement found that education, along with leadership and mentoring, facilitated engagement (Alexander et al., 2022).

A Europe-wide cross-sectional survey of hospital nurses in 2012 found that there were no academically educated nurses in Germany (Aiken et al., 2013). Since then, several initiatives have aimed to increase the rate of academic training, including the Act on Nursing Professions (Pflegeberufereformgesetz), which introduced a university-level degree as an equivalent alternative to vocational training, becoming effective in 2020 (Act on the Nursing Professions (Pflegeberufegesetz - PflBG), 2025). Results from a survey of a representative sample of German hospitals in 2023 indicated that approximately 80% of general acute care hospitals employed academically educated nurses. On average, each of these hospitals had around 15 (median six) academically educated nurses, demonstrating a relatively low number of academically qualified nurses per hospital (German Hospital Institute, 2024). A longitudinal survey of 14 German university hospitals showed that the proportion of academically educated nurses, working in direct patient care, increased from 2.14% in 2018 to 3.18% in 2024 (Pinnekamp et al., 2025). In contrast, the national nursing workforce survey of registered nurses in the U.S. indicated that nurses holding a Baccalaureate or master’s degree increased from 41.8% in 2015 to 51.5% in 2024 (Smiley et al., 2025). Additionally, Magnet® requires hospitals to support nurses’ professional development activities and mandates that nurses in certain positions, such as nurse managers, hold an academic degree (ANCC, 2024). Hence, nurses’ educational level as well as the educational requirements by Magnet® might explain the stronger integration of nursing staff in working with nurse-sensitive indicators.

Furthermore, the Magnet® model emphasizes contributions to nursing practice and the application of existing evidence (ANCC, 2025). This is consistent with reports from interviewees in U.S. Magnet® hospitals, who highlighted their institutions’ strong focus on evidence-based practice and continuous quality improvement goals. In a cross-sectional survey of nursing researchers in U.S. Magnet® hospitals, over 90% confirmed that their hospitals had implemented an evidence-based practice model and incorporated research findings into clinical practice (Speroni et al., 2020). In Germany, evidence-based nursing practice is supported by evidence-based expert standards, which are scientifically grounded guidelines for specific areas of care, such as the management of pressure injuries or falls (DNQP, 2025). The application of expert standards was also mentioned by German hospital interviewees; however, German interviewees highlighted further potential for strengthening evidence-based working. A study conducted in 2012 in German hospitals found that nurses generally held positive attitudes toward evidence-based practice, but implementation was considered challenging, for example, because nurses were not consistently informed about recent research (Köpke et al., 2013). This is also reflected by German interviewees. Literature access was described as important by all interviewees and simultaneously some interviewees from German hospitals reported hurdles in accessing literature.

### 4.3 Evaluation and dissemination of data-driven improvements of nursing practice

The differences in the academic level of nurses between the U.S. and Germany described above may also help explain the more systematic evaluation of implemented measures reported by interviewees from U.S. hospitals compared with those from German hospitals. This aspect corresponds to the third phase of a learning health system, in which performance data is collected to generate new knowledge. In addition, differences in the establishment of evidence-based nursing practice – which was also described above – between the two countries may contribute to this finding. As nursing research constitutes an important foundation of evidence-based nursing practice, its stronger integration into clinical practice may facilitate the systematic evaluation of practice changes and the dissemination of evidence-based improvements (Powers, 2020).

In Germany, nursing often lacks a culture of success that recognizes and communicates achievements (Hessen, 2019). This aligns with the findings from the German hospitals that described potential for improvement in identifying and communicating nurses’ successes. An integrative review on the impact of Magnet® found that it has a positive effect on organizational culture for nurses (Anderson et al., 2018). This is consistent with reports from the interviewees in U.S. Magnet® hospitals, who described recognizing and disseminating nurses’ successes in a systematic and multifaceted way.

### 4.4 Implications for future research

Although previous studies have explored facilitators and challenges for nurses’ engagement in quality improvement (Alexander et al., 2022; Mulkey, 2021), further research is needed to investigate barriers and enabling factors for the integration of nurse-sensitive indicators into everyday nursing practice. Strengthening this integration has the potential to improve nursing practice and enhance patient safety in both countries by enabling data-driven and systematic identification of areas for improvement.

The German hospital interviewees analyzed in this study were all affiliated with a twinning hospital from the U.S., experienced with Magnet® principles, and therefore familiar with engaging in quality improvement, nursing research, and the collection of nurse-sensitive indicators. But research should not be limited to hospitals engaged with Magnet® principles, as the use and impact of nurse-sensitive indicators are relevant for all hospitals.

### 4.5 Limitations

The following limitations need to be considered. This study focused on a purposive sample of U.S. Magnet® hospitals and German hospitals implementing the Magnet® model and having an U.S. Magnet® hospital as a twinning partner; therefore, the results are not generalizable but reflect the situation in hospitals with clear commitment to using data, improving work environment, nursing and quality. The relatively high proportion of interviewees in leadership roles, combined with an average work experience of 21 years, should be considered when interpreting the study findings, as these interviewees are likely to have greater familiarity with and place more emphasis on the strategic relevance of nurse-sensitive indicators. However, the inclusion of nurses with lower work experience and those not in leadership positions ensured that a broader range of perspectives was integrated, balancing the sample and supporting the relevance of the findings for both research and practice. Differences between the German and U.S. samples, including gender distribution and the proportion of interviewees still actively involved in clinical patient care, arose from the recruitment process and interviewees’ responses. While these factors may have influenced some perspectives, the main themes were consistent across the German and U.S. samples, supporting the relevance of the findings. The utilization of nurse-sensitive outcome indicators was assessed to identify areas for improving nursing practice. Neither the quality of those indicators nor other dimensions of the quality of nursing were explored (Gormley et al., 2024).

## 5 Conclusion

This qualitative study analyzed the utilization of nurse-sensitive indicators for improving nursing practice and patient care in U.S. Magnet® hospitals and German hospitals, being part of the Magnet4Europe study. Interviewees from U.S. hospitals, which had a larger basis of nurse-sensitive indicators, reported a more structured approach in using this data for quality improvement. In contrast, interviewees from German hospitals showed a high divergence in the utilization of nurse-sensitive indicators, highlighting the potential of strengthening the use of nurse-sensitive indicators in the future.

## Declaration of generative AI and AI-assisted technologies in the manuscript preparation process

During the preparation of this work the authors used ChatGPT in order to assist with reviewing English language and grammar. After using this tool, the authors reviewed and edited the content as needed and take full responsibility for the content of the published article.

## Funding source

Julia Köppen received salary support from the Bosch Health Campus during the conduct of this research. The funder had no role in the study planning, design, interpretation of the findings or write-up of the manuscript.

## Attachments

Appendix A: SRQR Checklist.

## CRediT authorship contribution statement

**Carolin Gurisch:** Writing – review & editing, Writing – original draft, Visualization, Project administration, Methodology, Investigation, Formal analysis, Data curation, Conceptualization. **Joan Kleine:** Writing – review & editing, Validation. **Julia Köppen:** Writing – review & editing, Methodology. **Claudia B. Maier:** Writing – review & editing, Supervision, Methodology.

## Declaration of competing interest

The authors declare that they have no known competing financial interests or personal relationships that could have appeared to influence the work reported in this paper.

## Data Availability

The data that support the findings of this study are not publicly available due to the sensitivity of the qualitative interview material and the confidentiality assurances given to participants. Data may be available from the corresponding author upon reasonable request.
